# A nomogram to predict the treatment benefit of perampanel in drug-resistant epilepsy patients

**DOI:** 10.3389/fneur.2023.1284171

**Published:** 2023-11-14

**Authors:** Chaofeng Zhu, Juan Li, Dazhu Wei, Luyan Wu, Yuying Zhang, Huapin Huang, Wanhui Lin

**Affiliations:** ^1^Department of Neurology, Fujian Medical University Union Hospital, Fuzhou, China; ^2^Fujian Key Laboratory of Molecular Neurology, Fuzhou, China; ^3^Department of Geriatrics, Fujian Medical University Union Hospital, Fuzhou, China

**Keywords:** epilepsy, perampanel, nomogram, predictive model, development and validation

## Abstract

**Objective:**

The objective of this study was to identify the factors that affect the efficacy of added perampanel for the treatment of drug-resistant epilepsy (DRE), and to develop a reliable nomogram to predict the benefit of this addition.

**Methods:**

A retrospective clinical analysis was conducted on DRE patients who received perampanel treatment and who were followed up for at least 6 months from January 2020 and September 2023 at the Epilepsy Center of Fujian Medical University Union Hospital. Data from January 2020 to December 2021 were used as development dataset to build model, while the data from January 2022 to September 2023 were used as validation dataset for internal validation. The predictive factors that affected the efficacy of perampanel as DRE treatment were included in the final multivariate logistic regression model, and a derived nomogram was established.

**Results:**

A total of 119 DRE patients who received perampanel treatment were included in this study (development datasets: *n* = 76; validation data: *n* = 43). Among them, 72.3% (*n* = 86) showed a 50% or greater reduction in seizure frequency after perampanel treatment. Of all the parameters of interest, sex, age, history of generalized tonic-clonic seizures, and the number of antiseizure medications were identified as significant predictors for estimating the benefit of adding perampanel for the treatment of DRE. A model incorporating these four variables was developed, and a nomogram was constructed to calculate the probability of benefit of adding perampanel using the model coefficients. The C-index of the predictive model was 0.838, and the validation C-index was 0.756. The goodness-of-fit test showed good calibration of the model (*p* = 0.920, 0.752 respectively).

**Conclusion:**

The proposed nomogram has significant clinical potential for predicting the probability of benefit of perampanel as DRE treatment. This nomogram can be used to identify DRE patients who could benefit from the early addition of perampanel to their treatment regimen.

## Highlights

- In the present study, a predictive model for predicting the probability of benefit of perampanel for DRE treatment was built based on the clinical factors of patients with epilepsy.
- The model proved to be well discriminated and calibrated, indicating excellent discriminative ability and general applicability.
- This model facilitates the process of distinguishing DRE patients with a high probability of benefit for the addition of perampanel treatment.

## Introduction

Epilepsy is a prevalent chronic neurological disorder afflicting over 70 million individuals worldwide (1, 2). This disorder is characterized by recurrent paroxysmal, transient, repetitive, and stereotyped seizures that exhibit notable clinical heterogeneity. Despite the availability of a plethora of antiseizure medications (ASMs), up to 30% of patients with epilepsy exhibit inadequate responses to treatment and develop drug-resistant epilepsy (DRE) (3). According to the International League Against Epilepsy (ILAE) working group, DRE is defined as failure to achieve sustained seizure freedom following the proper administration of two independent ASMs, either as monotherapy or combination therapy (4). Patients with DRE are at risk of premature mortality, injury, psychosocial dysfunction, and reduced quality of life (2). Therefore, there is a pressing need for novel and efficacious treatment modalities.

ASMs with innovative mechanisms of action represent potential avenues for seizure control in drug-refractory epilepsy. Activation of alpha-amino-3-hydroxy-5-methyl-4-isoxazolepropionic acid (AMPA) receptors has been implicated in seizure induction (5, 6). Preclinical research has demonstrated that AMPA receptor antagonists reduce or eliminate epileptiform activity *in vitro*, whereas blocking NMDA receptors is insufficient to abolish epileptiform discharges (7). Moreover, AMPA receptor activation is thought to participate in seizure synchronization and to facilitate the transition from epileptiform discharges to seizure activity (8). Consequently, the blockade of AMPA receptors may suppress seizures. Based on these premises, perampanel, a novel drug targeting AMPA receptors, has entered clinical trials (9). Perampanel is a highly selective and non-competitive antagonist of AMPA. In 2012, perampanel received approval for the adjunctive treatment of focal and generalized seizures in patients aged 12 years and older in Germany (10). In September 2019, it was approved as an adjunctive therapy for focal seizures (with or without secondary generalized seizures) in patients aged 12 years and older in China (11). The principal mechanism of action of perampanel is non-competitive binding to AMPA receptors on postsynaptic membranes, selectively inhibiting these receptors to decrease glutamatergic neurotransmission (5). As such, it inhibits glutamate-induced excitatory neurotransmission and exerts antiepileptic effects. Several randomized controlled trials of perampanel (including multiple phase II and III clinical trials) have demonstrated its superior efficacy compared to a placebo as an adjunctive therapy for refractory focal seizures, with a safe and well-tolerated dose range (4, 8, and 12 mg/day) (12–14). Nevertheless, a subset of DRE patients fail to improve their seizures following perampanel administration and experience adverse psychiatric effects, such as dizziness and drowsiness. Therefore, developing reliable methods to predict which patients with DRE may benefit from perampanel is of paramount importance.

Nomograms have recently been recognized as dependable instruments to create intuitive and simple graphical models that quantitatively predict the risk of clinical events. The need for integrated models to promote personalized medicine has been met, making it more convenient for clinicians to make prognosis predictions (15, 16). Nomogram models generate more precise and intuitive predictions than traditional assessment methods. These models are graphical tools based on regression models that quantify the risk of an event through various predictive factors. Nomograms are primarily employed to establish prediction models. By converting conventional statistical prediction models into visual graphics, nomograms can accurately predict the relationship between several variables and outcome indicators (17, 18). Presently, nomograms have been extensively employed in clinical settings. However, there is no nomogram model to predict the potential benefits of adding perampanel to the treatment of DRE patients.

In this study, our objective was to determine the clinical variables that significantly enhance seizure outcomes in patients after the administration of perampanel. Therefore, we constructed a nomogram to predict the probability of significant improvement in seizure outcomes following perampanel administration to support clinical decision-making.

## Materials and methods

### Study participants

This retrospective study aimed to collect the medical records of DRE patients who received adjunctive perampanel treatment at the Epilepsy Center of Fujian Medical University Union Hospital between January 2020 and September 2023. The inclusion criteria were as follows: (1) DRE patients who met the ILAE definition and received perampanel treatment; (2) patients with complete baseline seizure frequency records for at least 6 months before perampanel treatment initiation; (3) patients aged >14 years; (4) Patients with complete medical records; and (5) patients who were willing to participate in follow-up. The exclusion criteria were as follows: (1) irregular ASM intake; (2) patients with severe liver or kidney dysfunction; (3) patients with unclear medical records; (4) patients who underwent epilepsy-related surgery, vagus nerve stimulation, or ketogenic diet during perampanel treatment; and (5) patients with a follow-up time of <6 months.

In total of 119 DRE patients receiving adjunctive perampanel treatment met the eligibility criteria for this study. The Ethics Committee of Fujian Medical University Union Hospital approved this study, and informed consent for the use of medical records was obtained from all participants in compliance with the Helsinki Declaration. We confirm that we have read the Journal's position on issues involved in ethical publication and affirm that this report is consistent with those guidelines.

### Perampanel treatment regimen

Perampanel dose titration was conducted based on the clinical practice of our epilepsy center. The patients received once-daily perampanel before bedtime, starting at 2 mg/day. The dose was gradually increased by 2 mg every 2–4 weeks, based on the individual patient's clinical response and tolerability. The maintenance dose was determined according to the guidelines, drug interactions, and patient drug response. Physicians adjusted the dose at their discretion based on the patient's clinical response.

### Data collection

Data were obtained from the clinical records of patients and involved a baseline evaluation as well as follow-up assessments every 1–2 months following the administration of perampanel treatment. The following parameters were collected at baseline: demographic variables (including sex, age, body weight, body mass index [BMI], education years); clinical features (including seizure type, seizure frequency, video-EEG [VEEG], course of epilepsy); epilepsy type categorized by the origin of the epileptic focus in the brain (based on previous EEG, neuroimaging, and VEEG monitoring results); etiology; age of onset; medical history of generalized tonic-clonic seizures (GTCS), febrile seizures, traumatic brain injury, previous neurological disease; existence of psychiatric comorbidities, cognitive impairment (prior to the initiation of perampanel treatment); number of antiseizure medications (ASMs); and baseline seizure frequency. Baseline seizure frequency was defined as the frequency of seizures during the 6-month period before the initiation of perampanel.

Seizure frequency and response rates were assessed at every outpatient visit, typically every 1–2 months, based on clinical evaluations. Seizure frequency was evaluated based on patient and caregiver reports, as well as seizure records in a seizure diary collected during each clinical visit. Response was assessed at the final visit by comparing the seizure frequency to that at baseline; the patients who experienced a reduction in seizure frequency of 50% or more were classified as responders, while those with a reduction in seizure frequency of <50% or no improvement were categorized as non-responders.

### Comorbidities assessment

Cognitive function was assessed using the Montreal Cognitive Assessment (MoCA) and Mini-Mental State Examination (MMSE). Participants with MoCA scores ≥26 and MMSE scores ≥24 were classified as cognitively normal, whereas those with MoCA scores ≤25 or MMSE scores <24 were considered to have cognitive impairment. Anxiety and depression levels were estimated using the Self-rating Anxiety Scale (SAS) and Self-rating Depression Scale (SDS). To examine the influence of anxiety and depression on the study findings, the patients were stratified into two subgroups: those without anxiety or depressive symptoms (SAS < 50 and SDS < 53) and those with anxiety or depressive symptoms (SAS ≥ 50 or SDS ≥ 53), designated as no SAS/SDS and SAS/SDS, respectively.

### VEEG analysis

The VEEG data were scrutinized and classified as “normal EEG,” “abnormal background without epileptiform discharges,” or “epileptiform discharges” based on the original VEEG recordings or reports. The interictal VEEGs of all patients were recorded before perampanel administration.

### Brain MRI analysis

Magnetic resonance imaging (MRI) was performed for all patients to rule out structural abnormalities. The MRI reports were reviewed and categorized as “Negative” or “Positive.” “Positive” indicated that the MRI revealed structural abnormalities, including cerebral arteriovenous malformation, aneurysms, brain malformation, encephalomalacia and gliosis, partial cerebral parenchyma, hyperintense hippocampi, and focal cortical dysplasia. “Negative” referred to MRI findings without structural abnormalities.

### Statistical analysis

The data were analyzed using SPSS 26.0 software (IBM Corp.) and STATA 16 software. Numerical data are expressed as percentages, and continuous data are presented as mean ± standard deviation (SD). Univariate and multivariate logistic regression analyses were performed to identify factors that may influence DRE.

### Nomogram model construction and validation

Data from January 2020 to December 2021 were used as development dataset to build model, while the data from January 2022 to September 2023 were used as validation dataset for internal validation. Univariate logistic regression analysis was performed to identify the factors affecting the potential benefits of perampanel treatment for patients with DRE. Those factors with a *p-*value < 0.10 were included in the multivariate analysis. The independent factors were then determined using multivariate logistic regression analysis, with a backward stepwise approach based on the Akaike Information Criterion (AIC), to identify the most precise combination of useful factors for predicting the benefits of perampanel treatment for DRE.

Subsequently, a nomogram model was constructed based on the multivariate logistic regression model, with the aim of predicting the benefit probability of perampanel treatment. The performance of the nomogram model was evaluated using two main parameters: discrimination and calibration. Discrimination refers to the ability of a model to differentiate between patients who will and will not experience an event. The concordance index (C-index) and receiver operating characteristic (ROC) curve were used to evaluate the discriminative ability of the nomogram.

Calibration was used to assess the consistency between predicted and observed survival. A calibration plot was constructed to evaluate the calibration of the nomogram model. Finally, decision curve analysis (DCA) was applied to calculate the net benefits, thereby enabling an assessment of the performance of the model.

## Results

### Patient characteristics

In total of 119 patients diagnosed with DRE and treated with perampanel at our epilepsy center between January 2020 and September 2023 were included in this study after meeting the predetermined inclusion and exclusion criteria. The patients were an average age of 30.24 and 31.58 years, with a mean age of onset of 18.95 and 19.56 years, a mean disease duration of 11.71 and 12.44 years, and a mean education duration of 9.59 and 10.67 years. Of the patients enrolled in this study, 73.7 and 69.77% (56/76 and 30/43) experienced a significant reduction of 50% or more in seizure frequency after receiving perampanel treatment in the development and validation dataset. Additionally, 43.4% (33/76) and 44.2% (19/43) of patients had comorbid depression, 44.7% (34/76) and 44.2% (19/43) had comorbid anxiety, and 55.3% (42/76) and 58.1% (25/43) had comorbid cognitive impairment in the development and validation dataset. Table 1 presents the patient characteristics.

**Table 1:** Baseline characteristics of the investigated patients.

**Characteristic**	**Training set (*n =* 76)**	**Validation set (n = 43)**
**Gender**		
Male	38 (50.0)	21 (48.8)
Female	38 (50.0)	22 (51.2)
**Age, years**		
Mean ± Std. Deviation	30.24 ± 14.89	31.58 ± 15.45
**Weight**		
Mean ± Std. Deviation	62.18 ± 18.00	64.83 ±20.69
**BMI**		
Mean ± Std. Deviation	22.60 ± 4.90	23.60 ± 5.61
**Education years**		
Mean ± Std. Deviation	9.59 ± 4.21	10.67 ± 5.32
**Age of onset, years**		
Mean ± Std. Deviation	18.95 ± 14.08	19.56 ± 16.34
**Seizure type**		
Focal onset	34 (44.7)	22 (51.1)
Mixed onset	37 (48.7)	18 (41.9)
Generalized onset	5 (6.6)	3 (7.0)
**Seizure frequency**		
≥1 seizures per day	44 (57.9)	23 (53.5)
< 4 seizures per month	21 (27.6)	13 (30.2)
1–6 seizures per week	5 (6.6)	4 (9.3)
≤ 6 seizures per 6 months	6 (7.9)	3 (7.0)
**EEG**		
Normal	3 (3.9)	0
Abnormal background	8 (10.5)	5 (11.6)
Epileptiform discharges	65 (85.5)	38 (88.4)
**Course of epilepsy**		
Mean ± Std. Deviation	11.71 ± 10.07	12.44 ± 9.60
**Temporal lobe epilepsy**		
No	42 (55.3)	23 (53.5)
Yes	34 (44.7)	20 (46.5)
**Etiology**		
Structural	52 (68.4)	31 (72.1)
Others	24 (31.6)	12 (27.9)
**History of GTCS**		
No	44 (57.9)	30 (69.8)
Yes	32 (42.1)	13 (30.2)
**History of febrile seizures**		
No	66 (86.8)	38 (88.4)
Yes	10 (13.2)	5 (11.6)
**History of traumatic brain injury**		
No	70 (92.1)	40 (93.0)
Yes	6 (7.9)	3 (7.0)
**Number of ASM**		
Mean ± Std. Deviation	1.99 ± 0.841	2.12 ± 1.005
**Comorbid depression**		
No	43 (56.6)	24 (55.8)
Yes	33 (43.4)	19 (44.2)
**Comorbid anxiety**		
No	42 (55.3)	24 (55.8)
Yes	34 (44.7)	19 (44.2)
**Comorbid cognitive impairment**		
No	34 (44.7)	18 (41.9)
Yes	42 (55.3)	25 (58.1)
**Brain MRI**		
Negative	26 (34.2)	14 (32.6)
Positive	50 (65.8)	29 (67.4)
**Previous neurological disease**		
No	51 (67.1)	30 (69.8)
Yes	25 (32.9)	13 (30.2)

In our cohort, 14 patients had adverse reactions, including vertigo (*n* = 6), irritability (*n* = 2), weight gain (*n* = 2), somnolence (*n* = 2), and digestive system symptoms (*n* = 2). Most patients had no or could tolerate mild adverse reactions.

### Risk factors for DRE

To determine the potential factors that may influence the efficacy of perampanel treatment in patients with DRE, we performed univariate logistic regression analyses for each variable. Variables with a *p*-value <0.10 included sex, age, age at onset of first seizure, history of GTCS, and the number of antiseizure medications (ASMs) used (Table 2). These five variables with a *p*-value <0.10 were entered into the initial multivariable logistic regression analysis, and after eliminating irrelevant factors, four variables remained in the final logistic regression model: sex, age, history of GTCS, and number of ASMs (Table 3).

**Table 2:** Univariate logistic regression analysis of clinical candidate predictors.

**Variables**	**Univariate analysis**		***P***
	**OR**	**95% CI**	
Gender (Male vs. Female)	4.304	1.372 to 13.507	0.012
Age, years (continuous)	1.043	0.998 to 1.091	0.063
Weight, kg (continuous)	0.994	0.967 to 1.023	0.700
BMI (continuous)	0.985	0.889 to 1.092	0.778
Education years (continuous)	0.984	0.870 to 1.112	0.796
Age of onset, years (continuous)	1.059	1.003 to 1.119	0.040
**Seizure type**			0.287
Mixed onset vs. Focal onset	0.458	0.046 to 4.578	0.506
Generalized onset vs. Focal onset	1.071	0.103 to 11.130	0.954
**Seizure frequency**			0.928
< 4 seizures per month vs. ≥1 seizures per day	0.533	0.056 to 5.046	0.584
1–6 seizures per week vs. ≥1 seizures per day	0.500	0.048 to 5.224	0.563
< 6 seizures per 6 months vs. ≥1 seizures per day	0.800	0.037 to 17.196	0.887
**EEG**			0.797
Abnormal background vs. Normal	0.590	0.127 to 2.738	0.501
Epileptiform discharges vs. Normal	0.885	0.438 to 1.789	0.934
Course of epilepsy (continuous)	0.996	0.947 to 1.048	0.880
Temporal lobe epilepsy (Yes vs. No)	1.729	0.600 to 4.980	0.310
Etiology (Structural vs. Others)	1.105	0.365 to 3.349	0.860
History of GTCS (Yes vs. No)	4.000	1.188 to 13.474	0.025
History of febrile seizures (Yes vs. No)	0.480	0.120 to 1.917	0.299
History of traumatic brain injury (Yes vs. No)	0.692	0.117 to 4.105	0.686
Number of ASM (continuous)	0.558	0.301 to 1.035	0.064
Comorbid depression (Yes vs. No)	0.529	0.189 to 1.485	0.227
Comorbid anxiety (Yes vs. No)	0.986	0.353 to 2.751	0.978
Comorbid cognitive impairment (Yes vs. No)	0.769	0.273 to 2.171	0.620
Brain MRI (Positive vs. Negative)	0.771	0.256 to 2.321	0.644
Previous neurological disease (Yes vs. No)	1.667	0.528 to 5.265	0.384

**Table 3:** Multivariate logistic regression analysis of clinical candidate predictors.

**Variables**	**Univariate analysis**		***P***
	**OR**	**95% CI**	
Gender (Male vs. Female)	7.038	1.800 to 27.514	0.005
Age, years	1.049	1.005 to 1.106	0.046
History of GTCS (Yes vs. No)	4.223	1.052 to 16.950	0.042
Number of ASM	0.454	0.218 to 0.943	0.034

### Nomogram model development and validation

**Figure 1:**
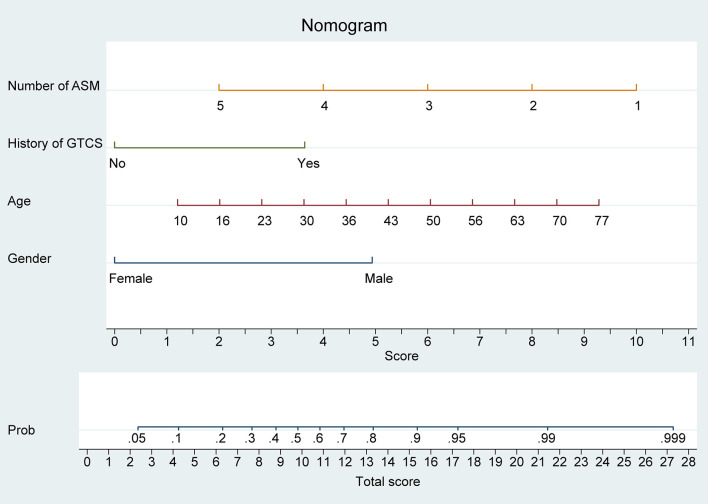
Nomogram model for predicting the efficacy of perampanel treatment in drug-resistant epilepsy (DRE) patients. The nomogram model integrates four predictive factors, including the number of anti-seizure medications (ASMs), history of generalized tonic-clonic seizures (GTCS), age, and sex. Each predictive factor is assigned a score based on its contribution to the overall model and the sum of these scores is used to calculate the predicted probability of benefiting from perampanel treatment.

We developed a model based on the results of multivariate logistic regression, which incorporated four key features. Subsequently, we plotted a nomogram (Figure 1) using the coefficients derived from the model to calculate the likelihood of benefit from adding perampanel for DRE treatment. The number of ASMs was allocated the highest weighting in the nomogram, followed by patient age, while the history of GTCS had the smallest impact on the benefit probability.

Our nomogram provides a convenient and accurate tool for predicting the likelihood of benefit from adding perampanel treatment in patients with DRE. For individual DRE patients, the position of each variable on the corresponding axis was determined, and the scores for each variable were summed to obtain a total score. The total score axis was used to estimate the benefit likelihood.

**Figure 2:**
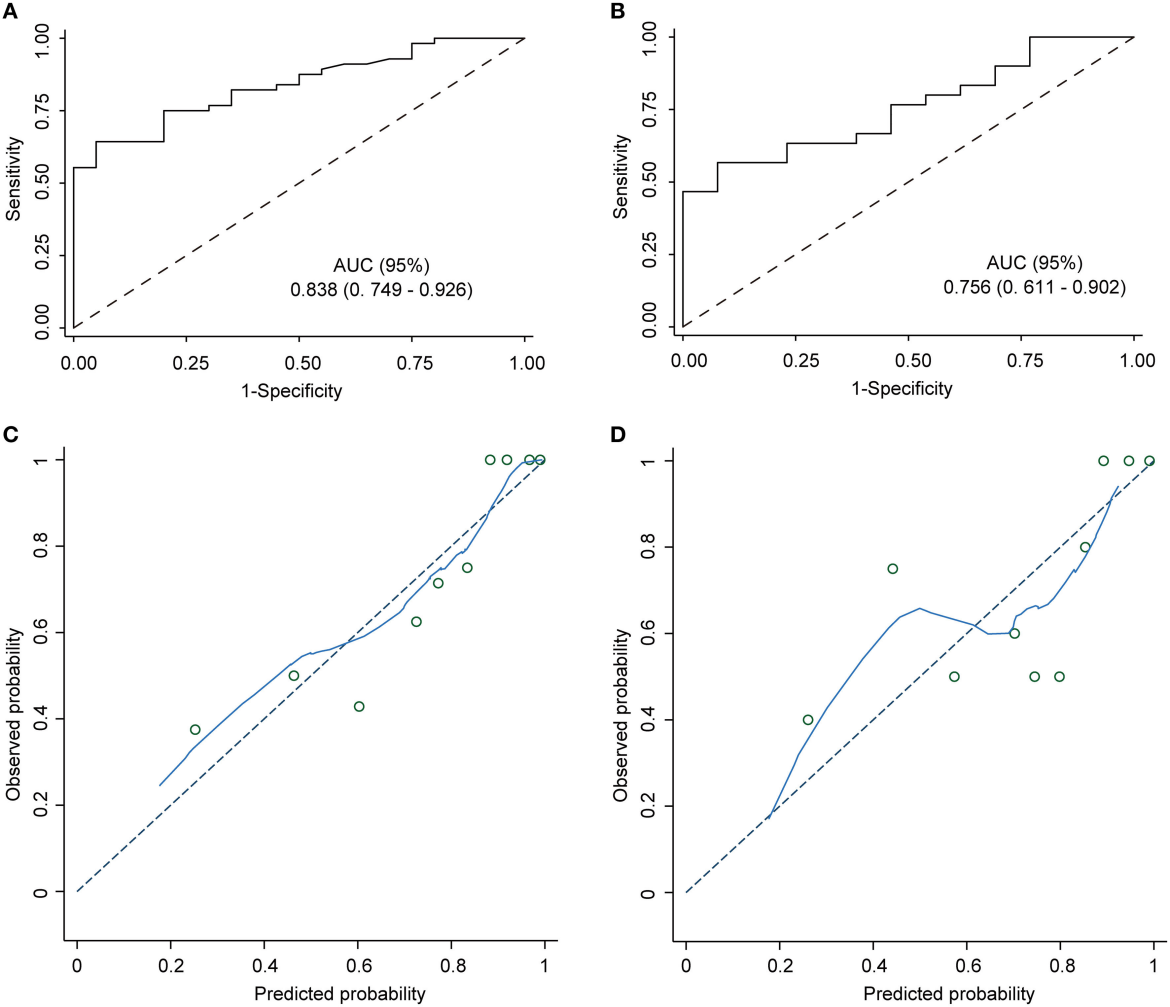
Nomogram model ROC and calibration curve. ROC curves of the predictive model in the development cohort and the validation cohort. Area under the ROC curve **(A)** shows the predictive ability of the model in the training cohort, and area under the ROC curve **(B)** validates the predictive ability of the model. ROC: receiver operating characteristic. The ROC curve plots the true positive rate (sensitivity) against the false positive rate (1 - specificity) at various classification thresholds. The diagonal dashed line represents the performance of a random guess, whereas the solid line represents the performance of the nomogram model. The closer the solid line is to the upper left corner of the plot, the better the model's discriminatory ability. **(C)** The nomogram calibration curve displays the predictive performance of the nomogram model in the training cohort. **(D)** The validation plot illustrates the distribution of the nomogram model's performance. The x-axis shows the predicted probability of the outcome, while the y-axis shows the actual probability of the outcome. The diagonal dashed line indicates perfect calibration, while the solid line represents the actual performance of the nomogram model.

**Figure 3:**
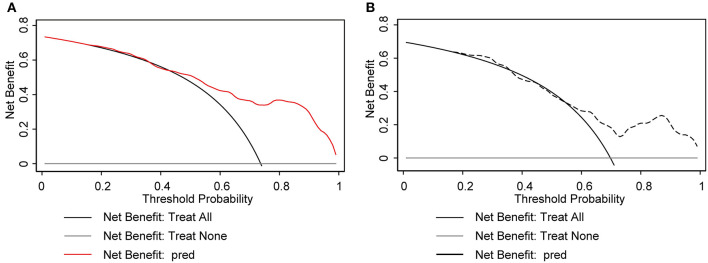
Decision curve analysis (DCA) plot for the nomogram model. The x-axis represents the threshold probability and the y-axis represents the net benefit. The gray solid line represents the net benefit of the “no-treatment” strategy, while the black solid line represents the net benefit of the “treat-all” strategy. The solid red line and black dotted line represents the net benefit of the nomogram model. The DCA plot in the development cohort **(A)** and the validation cohort **(B)** demonstrates that the nomogram model has a superior net benefit compared to the “treat-all” and “no-treatment” strategies across the range of threshold probabilities, indicating its potential usefulness as a clinical decision-making tool.

The nomogram demonstrated excellent accuracy in predicting the likelihood of benefit from adding perampanel treatment in DRE patients, with a C-index of 0.838 (95% confidence interval [CI], 0.749–0.926) in the development dataset and a C-index of 0.756 in the validation dataset (Figures 2A, B). The calibration curves for the development and validation dataset showed a high consistency between the predicted and observed outcomes (Figures 2C, D). The Hosmer-Lemeshow (H-L) goodness-of-fit test *p*-values were 0.920 and 0.752, respectively, indicating optimal calibration. The DCA demonstrated that our nomogram model also displayed a higher net benefit (Figure 3).

## Discussion

As per the ILAE's standardized definition, DRE is characterized by the failure to achieve sustained seizure freedom following appropriate trials of two tolerated, suitably selected, and used ASMs, either in monotherapy or in combination therapy (4). Studies have reported DRE incidence rates ranging from 15 to 34% (19, 20). Recent studies suggest that adjunctive perampanel therapy can effectively reduce seizure frequency in patients with DRE (6, 21, 22). Nevertheless, not all patients with DRE benefit from perampanel adjunctive therapy. Some studies have reported instances of no significant improvement in seizure frequency and even adverse psychiatric effects due to perampanel (6, 23). Identifying DRE patients with a high probability of benefiting from perampanel adjunctive therapy and administering it early in their disease course may result in improved quality of life and early benefits. Therefore, identifying patients with DRE who are most likely to benefit from perampanel adjunctive therapy is crucial.

Our observational investigation revealed a noteworthy discovery, wherein 72.3% of patients (*n* = 86) exhibited a positive response over the entire duration of observation, surpassing the typical range of efficacy reported in randomized controlled trials on perampanel, which typically register efficacy rates between 26 and 56% (24). A clinical practice conducted in Germany, based on 6 months of observation, indicated that 46% of patients responded to treatment, evincing a reduction of at least 50% in seizure frequency (25). Ishikawa et al.'s retrospective study demonstrated a response rate of 52.3% to perampanel therapy (26). The divergence in the efficacy results may be explained by the more treatment-resistant population in prior studies. Nevertheless, these findings underscore the efficacy of perampanel as an add-on therapy for patients with refractory epilepsy, which undoubtedly holds immense promise.

Variations in follow-up periods preclude comparisons of retention rates across distinct studies. However, our present study's retention rate of 81.60% in development dataset falls within the range of 44–89% reported by Lattanzi et al. (27), signifying the drug's good tolerability and efficacy. The effective dose of perampanel ranged from 2 to 12 mg/day. Notably, the final mean dose of perampanel in our study for all patients was 5.447 ± 2.18 mg/day in development dataset, which is lower than the mean doses reported in two other real-world studies (6.03 ± 2.43 mg/day and 6.3 ± 3 mg/day, respectively) (28, 29). This variation can be attributed to demographic and anthropometric differences, including geographical location, population distribution, race, and body weight. A Korean study further demonstrated that Asians require lower perampanel doses (30). Additionally, a comparative study of perampanel efficacy in Asians and non-Asians showed that Asians exhibit poor tolerability to high doses (10–12 mg/day) (31). A slow titration regimen (incrementally increasing the dose by 2 mg every 2–4 weeks) could be a potential solution to enhance retention rates, as reflected significantly in this study.

Our results demonstrate that several factors significantly influence the likelihood of perampanel therapy benefitting patients with DRE. These factors include sex, age, history of GTCS, and the number of ASMs taken. These factors were incorporated into a logistic regression model. Notably, the number of ASMs taken exhibited the highest predictive weight in estimating the benefit of perampanel therapy. Furthermore, patient age and sex also exhibited significant predictive potential in terms of the probability of treatment benefit, with older patients and male patients demonstrating a higher likelihood of benefit. The presence of GTCS had a predetermined value in predicting the development of DRE while also playing a critical role in predicting clinical outcomes; patients with a history of GTCS exhibited a superior clinical response to perampanel therapy.

Our analysis also indicates that patients who have previously taken fewer ASMs have a more favorable clinical response. These results parallel those observed in the context of lacosamide and lamotrigine in patients in the early stages of treatment (32). Accordingly, perampanel therapy exhibits a response pattern that aligns with that of other ASMs. Notably, in Vicente Villanueva's study on perampanel therapy for focal epilepsy, early perampanel adjunctive therapy was found to be more effective than extensive ASM use before perampanel initiation (25). Similar results were obtained in patients with idiopathic generalized epilepsy (33). Collectively, these findings suggest that perampanel is more effective as an early adjunctive therapy for patients with DRE. Our results represent a valuable contribution to the prediction of treatment efficacy with the addition of perampanel to the treatment of DRE. We postulate that the reason underlying these findings is that the success rate of seizure control after the first ASM failure declines with the introduction of subsequent ASMs.

Our study findings revealed that male patients exhibited a superior clinical response to perampanel administration. This result is in contrast with the findings reported by Vazquez et al. (34), who documented a better clinical response in females receiving perampanel. Nonetheless, no significant statistical disparity existed in the number of male and female patients who attained seizure freedom (34). Moreover, studies have established that sex does not influence the efficacy of perampanel, as evidenced by a prior sub-analysis of a phase III randomized clinical trial that found no sex-based disparity in the efficacy and tolerability of perampanel (35). Nevertheless, this discrepancy may stem from limitations in sample size, thus necessitating the conduct of larger-scale studies to validate whether sex is associated with the efficacy of perampanel adjunct therapy in patients with DRE.

Our study outcomes suggest that patients of advanced age and those with a history of GTCS exhibit a superior clinical response to perampanel. GTCS history has long been regarded as an independent risk factor for DRE and likely validates the rationale behind perampanel as an adjunct treatment for this patient group. Similarly, previous studies revealed that epilepsy patients aged ≥65 years showed a superior clinical response to perampanel (24), concurring with our study findings. Notably, all our patients had DRE, and increased patient age correlated positively with the probability of perceiving benefits from adding perampanel treatment. Further studies are necessary to decipher the mechanisms underlying this association.

The salient aspect of our study was the development of a clinical prediction model to forecast the probability of DRE patients experiencing benefits from perampanel adjunct therapy, characterized by adequate discrimination and calibration. However, limitations such as inaccurate self-reporting of seizure frequency and inadequate attention to adverse reactions, albeit alongside extensive research on perampanel adjunct treatment tolerability, underscore the need for caution in generalizing the results. Thus, it is imperative to conduct long-term real-world studies in the future.

## Conclusion

Our study established the efficacy of perampanel in minimizing seizure frequency in patients with DRE and devised a clinical prediction model to predict the probability of DRE patients benefitting from perampanel adjunct therapy. Sex, age, history of GTCS, and the number of ASMs remain essential predictive factors for this beneficial effect. Deploying predictive tools facilitates the identification of patients with DRE who are likely to benefit from adding perampanel adjunct therapy. Consequently, the early administration of perampanel has immense potential for enhancing treatment outcomes in such patients.

## Data availability statement

The raw data supporting the conclusions of this article will be made available by the authors, without undue reservation.

## Ethics statement

The studies involving humans were approved by the Ethics Committee of Fujian Medical University Union Hospital. The studies were conducted in accordance with the local legislation and institutional requirements. Written informed consent for participation in this study was provided by the participants' legal guardians/next of kin.

## Author contributions

CZ: Conceptualization, Data curation, Formal analysis, Methodology, Software, Validation, Visualization, Writing—original draft. JL: Data curation, Formal analysis, Methodology, Software, Investigation, Writing—review & editing. DW: Data curation, Formal analysis, Investigation, Methodology, Software, Writing—review & editing. LW: Data curation, Formal analysis, Investigation, Methodology, Writing—review & editing. YZ: Formal analysis, Investigation, Methodology, Writing—review & editing, Conceptualization, Resources, Supervision, Validation. HH: Conceptualization, Formal analysis, Investigation, Resources, Supervision, Writing—review & editing, Funding acquisition, Project administration. WL: Conceptualization, Funding acquisition, Investigation, Project administration, Writing—review & editing.
